# Draft Genome Sequence of Streptococcus agalactiae TA B490, a Multidrug-Resistant Strain Isolated from Bovine Mastitis in Argentina

**DOI:** 10.1128/MRA.01429-20

**Published:** 2021-02-04

**Authors:** J. S. Cadona, L. B. Hernandez, R. Lorenzo, E. Bottini, A. V. Bustamante, A. M. Sanso

**Affiliations:** a Laboratorio de Inmunoquímica y Biotecnología, Centro de Investigación Veterinaria de Tandil, CONICET-CIC-UNCPBA, Facultad de Ciencias Veterinarias, Universidad Nacional del Centro de la Provincia de Buenos Aires, Tandil, Argentina; b Centro de Investigación Veterinaria de Tandil, CONICET-CIC-UNCPBA, Facultad de Ciencias Veterinarias, Universidad Nacional del Centro de la Provincia de Buenos Aires, Tandil, Argentina; c Laboratorio de Microbiología Clínica y Experimental, Centro de Investigación Veterinaria de Tandil, CONICET-CIC-UNCPBA, Facultad de Ciencias Veterinarias, Universidad Nacional del Centro de la Provincia de Buenos Aires, Tandil, Argentina; University of Rochester School of Medicine and Dentistry

## Abstract

Streptococcus agalactiae is a bovine pathogen that causes intramammary infections. For humans, S. agalactiae is a leading cause of neonatal death and an emerging pathogen in adults. Here, we present the draft genome sequence of S. agalactiae TA B490, a multidrug-resistant strain isolated from bovine mastitis in Argentina.

## ANNOUNCEMENT

Streptococcus agalactiae is an important pathogen that causes clinical and subclinical mastitis in cattle, which has impacts on animal health and dairy production (1). It is also a human pathogen that causes serious infections, mainly in neonates, the elderly, and immunosuppressed people (2). In the framework of a project that includes the comparative genomic analysis of S. agalactiae strains and in order to identify genomic regions that could be predictive virulence markers, the complete genome of an Argentine bovine strain of S. agalactiae was sequenced.

S. agalactiae TA B490, a multidrug-resistant serotype II strain, was recovered from a cow with mastitis living in a dairy farm located in one of the largest milk-producing regions of Argentina, the Cuenca Mar y Sierras (area pampeana, Tandil). A milk sample was collected under aseptic conditions, immediately refrigerated at 4°C, and subjected to bacteriological analysis within 24 h after collection. A loopful of the milk sample was streaked onto Trypticase soy agar (TSA) enriched with 5% bovine blood, and plates were incubated at 37°C under microaerophilic conditions. Subsequently, the plates were examined for colony morphology, pigmentation, and hemolytic characteristics after 24 to 48 h. Presumptive colonies of *Streptococcus* species were selected and streaked onto a slant agar for 24 h for biochemical tests and Gram staining. Catalase, NaCl, bile esculin, Christie-Atkins-Munch-Peterson (CAMP), hippurate hydrolysis, and sorbitol tests were carried out according to the protocol described by the National Mastitis Council (3). To confirm the species identification, a region of the monocopy regulatory gene *dltR* specific to S. agalactiae was amplified by PCR according to the method described by Lamy et al. (4). Since an amplification product was obtained, we verified that it was an S. agalactiae strain. The strain presented a multidrug resistance profile (with resistance to kanamycin, clindamycin, pirlimycin, erythromycin, and tetracycline), using a disc diffusion method according to the Clinical and Laboratory Standards Institute instructions (5).

DNA was extracted from an overnight culture (obtained from a TSA plate enriched with 5% bovine blood and incubated at 37°C under microaerophilic conditions) using a Wizard genomic DNA extraction kit (Promega, USA) following the manufacturer’s guidelines. DNA was quantified using a NanoDrop 2000c spectrophotometer (Thermo Fisher Scientific, Waltham, MA, USA), and the integrity was further checked using 1% agarose gel electrophoresis.

The genome sequence was obtained using a whole-genome shotgun strategy with Illumina MiSeq technology (Omega Bioservices, Norcross, GA, USA). A HyperPrep kit (Kapa Biosystems, Wilmington, MA, USA) was used for whole-genome library construction. The libraries were quantified and qualified using the D1000 ScreenTape on an Agilent 2200 TapeStation instrument and were normalized and pooled for multiplexed sequencing on a MiSeq sequencer (Illumina, San Diego, CA, USA) using the paired-end 300-bp run format. The total number of sequencing reads obtained was 1,734,640 reads with an average length of 300.72 bp.

Sequencing data were uploaded to the Galaxy Web platform (6), and we used the public server (https://usegalaxy.org) to perform the assembly. Illumina sequence quality was first checked using FastQC v0.72 (http://www.bioinformatics.babraham.ac.uk/projects/fastqc), and trimming was performed using Trim Galore! v0.6.3 (https://www.bioinformatics.babraham.ac.uk/projects/trim_galore). Sequencing reads were then assembled *de novo* by SPAdes v3.12.0, and k-mer values were chosen automatically (7). The assembly was run with careful correction set on and coverage cutoff set off. The genome was assembled into 143 contigs with an *N*_50_ value of 39,265 bp and a maximum contig length of 104,508 bp. The genome is approximately 2.3 Mb long, with a G+C content of 35.64%. Coverage was checked by remapping the reads against the assembled contigs using BBMap v38.86 (8) giving an average coverage depth of 167.35×. Default parameters were used for mapping (e.g., k = 13, maxindel = 16000, bwr = 0, bw = 0, minhits = 1, trimq = 6, kfilter = 0, maxsites = 5). For submission, sequences smaller than 200 bp were removed. Assessment of assembly quality and completeness using BUSCO (9) indicated that 98.5% of the single-copy orthologs used for the assessment were present (complete, 98.5% [single, 98.5%; duplicated, 0.0%]; fragmented, 0.2%; missing, 1.3%; gene number, 402 genes). BUSCO (Galaxy v4.1.2) was run in genome mode using *Lactobacillales* as the closest lineage, the E value cutoff set to 0.01, and the candidate region limit set to 3; AUGUSTUS species parameters were automatically selected according to the predefined defaults.

For genome annotation, we used NCBI automatic annotation by the NCBI Prokaryotic Genome Annotation Pipeline (PGAP) (10, 11). A total of 2,195 coding genes, 55 tRNA genes, and 16 rRNA genes were predicted, with 11 partial sequences for 16S rRNA and 1 partial sequence for 5S rRNA.

The genome assembly was also examined using the ResFinder Web server v4.0 (12, 13). Resistances to lincomycin (clindamycin), macrolides (erythromycin), aminoglycosides (streptomycin), and tetracycline (tetracycline) were predicted on the basis of *ermB* (same resistance mechanism for the first two), *ant(6)-Ia*, and *tetO*, respectively.

*In silico* multilocus sequence typing (MLST) was performed by aligning Illumina reads with reference sequences from the S. agalactiae PubMLST database (https://pubmlst.org/sagalactiae). A new allele was found at the *sdhA* locus, identified as *sdhA*-153. Consequently, the isolate belonged to a new sequence type (ST), ST1640.

These sequence data constitute the first genomic information on an Argentine strain of S. agalactiae of bovine origin, and they report the presence of a previously undetected clone. These observations, together with comparative genomic studies, will provide better knowledge of the genetic elements that might offer targets for the development of diagnostic tests, as well as antimicrobial treatment options.

### Data availability.

This whole-genome shotgun project has been deposited in DDBJ/ENA/GenBank under the accession number JACZIC000000000. The version described in this paper is the first version (accession number JACZIC000000000.1). The BioProject accession number in GenBank is PRJNA666902. The SRA accession number is SRX9366858.
